# Probiotics regulate the intestinal microbiome to promote growth in juvenile *Salmo trutta fario*

**DOI:** 10.1038/s41598-026-35054-y

**Published:** 2026-01-11

**Authors:** Jianshe Zhoua, Kuankuan Leia, Peng Zhang, ZhuangZhuang Wang, Wanliang Wang

**Affiliations:** 1 Institute of Aquatic Sciences, Xizang Autonomous Region Academy of Agricultural and Animal Husbandry Sciences, Lasa, 850032 China; 2 Key Laboratory of Fishery and Germplasm Resources Utilization of Xizang Autonomous Region, Lasa, 850032 China; 3 School of Ecology and Environment, Xizang University, Lhasa, China

**Keywords:** Probiotics composition, Intestinal microbiome, *Salmo trutta*, EM probiotics, Microbiology, Zoology

## Abstract

Probiotics improve aquaculture growth and immunity, but their dosage effects on gut microbes remain unclear. This study evaluated the impact of different levels of Effective Microorganisms (EM) probiotics on growth performance and intestinal microbiota of Juvenile *Salmo trutta fario*. Dietary EM supplementation significantly enhanced growth, with the medium-dose group (0.5% EM) exhibiting the best performance. Dietary EM supplementation significantly enhanced growth, with the medium-dose group (0.5% EM) showing the best performance. EM influenced hepatic antioxidants, with the medium-dose group similar to the control, and altered serum biochemistry, particularly in medium and high-dose groups, while most hematological parameters remained unchanged. Furthermore, 0.5% EM reduced beta diversity and inter-individual variation in the gut microbiota, suggesting enhanced microbial stability. EM supplementation altered the relative abundance of beneficial genera, such as *Lactobacillus*, *Bifidobacterium*, and *Faecalibacterium*, in a dose-dependent manner. Co-occurrence network analysis revealed several probiotic genera as potential keystone taxa, suggesting their crucial roles in maintaining the stability and function of the intestinal microbiota. Overall, EM exhibited clear benefits for growth and development, with 0.5% showing superior effects over 0.1% and 1%, emphasizing the importance of probiotic dosage for fish health and providing a basis for optimizing EM application in *Salmo trutta* aquaculture.

## Introduction

*Salmo trutta* *fario* belongs to the order Salmoniformes, family Salmonidae, subfamily Salmoninae. It is a cold-water salmonid of high economic value in the Tibet Autonomous Region, where it has become a dominant aquaculture species following its introduction to Yadong County by the British in the early 20th century^1–4^. It is also listed as a second-class protected aquatic species in the region^4^. After long-term domestication and ecological adaptation, *Salmo trutta fario* has become widely cultured due to its tender meat and high nutritional quality^5–7^. However, the rapid expansion of aquaculture in recent years has led to increasingly frequent health problems, particularly those associated with disruptions of intestinal microecological balance, which now constrain the sustainable development of *Salmo trutta* farming^8^. The gut microbiota plays central roles in nutrient absorption, immune regulation, and pathogen resistance^9–11^. Among these microbial communities, probiotics have been widely demonstrated to modulate intestinal microecology and enhance host immunity, making them an important strategy for mitigating disease and improving the sustainability of aquaculture systems^9,12^.

Probiotics are beneficial and biologically active microorganisms capable of colonizing the intestines of aquatic animals or improving water quality^13^. They can enhance the growth performance and immune capacity of aquatic species while improving the culture environment, without causing adverse effects to consumers^2,14^. Studies have shown that the most commonly used probiotics in aquaculture belong to genera such as *Lactococcus*, *Lactobacillus*, *Enterococcus*, and *Saccharomyces*^12,15^. These probiotic strains not only help regulate the composition of the host gut microbiota but also inhibit the colonization of pathogenic microorganisms through competitive exclusion, thereby effectively reducing disease incidence. For example, a compound probiotic containing *Bacillus subtilis* and *Lactobacillus paracasei* significantly enhanced growth and disease resistance in *crucian carp*^13^. Likewise, dietary *Bacillus coagulans* supplementation improved growth rate and digestive efficiency in turbot^16–18^. Numerous studies have demonstrated that probiotics can suppress pathogenic microbes, enhance growth performance, and strengthen immune responses by stimulating both mucosal and systemic immunity, thereby alleviating intestinal inflammation and improving disease resistance in fish^19^ Therefore, probiotics are widely regarded as a promising green alternative to antibiotics, playing a vital role in advancing sustainable, efficient, and health-oriented aquaculture practices^11,15,18,20^.

Although probiotics are widely applied to enhance growth performance and overall health in aquatic organisms, numerous studies have shown that increasing the dosage does not always lead to improved outcomes. Instead, identifying an optimal dosage is essential to fully realize their benefits^9,19^. The appropriate dosage of probiotics is a key factor determining their effectiveness in aquaculture^21,22^. In our previous study^23,24^, we evaluated the effects of several commonly used probiotics such as Effective Microorganisms (EM)probiotics, *Lactobacillus*, *Bacillus subtilis* and *Bacillus* licheniformis added to basal diets on the growth performance and health status of *Salmo trutta fario*. The results showed that EM probiotics produced the most pronounced positive effects, significantly enhancing fish growth while promoting intestinal and immune health. However, the optimal EM supplementation level for juvenile *Salmo trutta fario* remains unclear, and the associated shifts in intestinal microbial communities under different EM dosages are still poorly understood. Therefore, this study aims to systematically investigate the effects of dietary EM probiotics at varying concentrations on the growth performance and gut microbiota of juvenile *Salmo trutta fario*. By employing high-throughput sequencing, we examine how EM supplementation influences microbial diversity, community structure, and co-occurrence patterns in the gut. The findings are expected to shed light on the mechanisms through which probiotics regulate intestinal homeostasis and to provide a theoretical and practical basis for optimizing probiotic use in aquaculture.

## Materials and methods

### Fish culture and experiment design

The experiment was conducted using cylindrical tanks with a diameter of 90 cm, a height of 60 cm, and a water depth maintained at 45 cm. Throughout the experimental period, a continuous flow-through water system and aeration pumps were employed to ensure adequate oxygenation. Water temperature was maintained between 12 and 13 °C, and dissolved oxygen levels were kept at 6–7 mg/L. The feeding trial was carried out from December 23, 2023, to March 1, 2024, at the Yarlung Zangbo River Fishery Resources Breeding Base, located in Chengguan District, Lhasa City, Xizang Autonomous Region. Prior to the trial, all juvenile *Salmo trutta fario* were acclimated for two weeks to reduce potential stress and allow them to adapt to the experimental conditions. This was followed by a 70-day feeding trial. All test fish were obtained from the Yarlung Zangbo River Fishery Resources Breeding Base under the Xizang Academy of Agricultural and Animal Sciences. Only healthy individuals with intact body surfaces and similar body sizes were selected for the trial. The initial phenotypic parameters of the juvenile *Salmo trutta* are shown in Table 1.

**Table 1:** Phenotypic growth data of triploid rainbow trout under different stocking densities.

Group	Body weight (g)	Body length (mm)	Length (mm)
CK	66.6 ± 0.29 a	93.8 ± 1.00 a	105 ± 1.13 a
L	67.4 ± 0.25 a	95.0 ± 0.90 a	110 ± 0.97 a
M	69.2 ± 0.33 a	93.3 ± 0.96 a	109 ± 10.4 a
H	68.7 ± 0.28 a	94.0 ± 0.87 a	107 ± 0.97 a

The same letter indicates that the difference is not significant (*p*>0.05); Different letters of the peer indicate significant differences (*p*<0.05). CK, Control group; L, Low-dose group; M, Medium-dose group; H, High-dose group.

### Feed composition and probiotic preparation

A commercial formulated feed specifically designed for rainbow trout was provided by Sichuan Zhonglian Hechuang Biotechnology Co., Ltd. The basal diet contained the following main nutritional components: crude protein ≥ 50.0%, crude fat ≥ 12.0%, crude fiber ≤ 3.0%, crude ash ≤ 15.0%, total phosphorus ≥ 1.4%, lysine ≥ 3.0%, and moisture < 12.5%. Prior to use, the feed was sterilized by high-temperature steam treatment at 100 °C for 30 min. The EM probiotic suspension was diluted with sterile distilled water and uniformly sprayed onto the sterilized feed. The amount of EM applied was adjusted to obtain final dietary treatments containing 0% (CK), 0.1% (L), 0.25% (M), and 0.5% (H) EM. The treated feed was stored at 4 °C in the dark and was used within 24 h to ensure freshness and probiotic viability. The daily feeding rate was set at 1% of the fish’s body weight, delivered in two equal portions each morning and evening.

### Sample collection and processing

After 70 days of feeding, all fish were subjected to a 24-hour fasting period. Three juvenile *Salmo trutta fario* were randomly selected from each tank, with a total of nine fish per group. Fish were anesthetized using MS-222 (ethyl 3-aminobenzoate methane sulfonate, 100 mg/L; Beijing Green Hengxing Biotechnology Co., Ltd.), and intestinal contents were collected for microbial DNA extraction. The samples were immediately stored at −80 °C. Blood samples were collected via the caudal vein using a 1.0 ml sterile syringe. A portion of each sample was placed in EDTA-K2 anticoagulant tubes for hematological analysis. The remaining blood was transferred into RNase-free 1.5 ml centrifuge tubes and stored at 4 °C overnight. The samples were then centrifuged at 3000 r/min for 15 min at 4 °C to obtain the serum, which was aliquoted and stored at −80 °C (Thermo Fisher Scientific) for biochemical analyses. All dissection procedures were conducted under aseptic conditions using autoclaved surgical instruments in a laminar flow hood. Body weight, liver weight, and viscera weight were recorded for each fish to calculate growth performance indices. Liver tissues were immediately snap-frozen in liquid nitrogen and stored at −80 °C for antioxidant parameter analyses. For biochemical assays, approximately 1 g of liver tissue was homogenized in 9 ml of physiological saline, followed by centrifugation at 3000 r/min for 10 min. The resulting supernatant was collected and kept at 4 °C until further analysis.

### Determination of hepatic antioxidant, hematological, and serum biochemical parameters

Hepatic antioxidant parameters included malondialdehyde (MDA), superoxide dismutase (SOD), catalase (CAT), and reduced glutathione (GSH). All assays were performed using commercial kits provided by Jiangsu Meimian Industrial Co., Ltd. (Jiangsu, China), strictly following the manufacturer’s instructions. Hematological parameters were measured using an automated veterinary hematology analyzer (TEK-VET3). The measured indices included white blood cell count (WBC), absolute lymphocyte count (Lym), absolute intermediate cell count (Mid), absolute granulocyte count (GR), platelet count (PLT), mean platelet volume (MPV), plateletcrit (PCT), platelet distribution width (PDW), large platelet cell ratio (P-LCC), red blood cell count (RBC), mean corpuscular volume (MCV), hematocrit (HCT), and hemoglobin concentration (HGB). Serum biochemical parameters included alanine aminotransferase (ALT), aspartate aminotransferase (AST), alkaline phosphatase (ALP), glucose (GLU), total protein (TP), albumin (ALB), high-density lipoprotein cholesterol (HDL), low-density lipoprotein cholesterol (LDL), triglycerides (TG), and total cholesterol (TC). All measurements were conducted using standardized procedures and assay kits provided by Jiangsu Meimian Industrial Co., Ltd.

### DNA extraction, 16 S rRNA gene amplification, and sequencing

DNA was extracted from the intestinal content samples using the cetyltrimethylammonium bromide (CTAB) method. The purity and concentration of the extracted DNA were measured using a NanoDrop 2000 spectrophotometer (Thermo Scientific, Wilmington, DE, USA), and DNA integrity was assessed by 1% agarose gel electrophoresis. The V3–V4 hypervariable region of the bacterial 16 S rRNA gene was amplified using the primers 341 F (5’-CCTAYGGGRBGCASCAG-3’) and 806R (5’-GGACTACNNGGGTATCTAAT-3’). The resulting PCR products were purified and used to construct sequencing libraries with the TruSeq DNA PCR-Free Sample Preparation Kit (Beckman Coulter Life Sciences, USA). High-throughput sequencing was performed on the Illumina NovaSeq 6000 platform. Raw sequencing data were quality-filtered using FASTP (v0.33) to remove low-quality reads. After quality filtering of the raw data, high-quality clean data were obtained for subsequent analysis. The clean data were demultiplexed separately according to their unique barcodes. Amplicon sequence variations (ASVs) were obtained using a standard denoising pipeline. ASVs were obtained using the DADA2 plugin in the QIIME2 software (version 2023.5), after which the ASV abundance table was constructed. ASVs were annotated using the SILVA database (version 138). ASVs with low abundance (< 10 reads) were removed. Three replicates were used to reduce sampling bias. The ASVs table was then rarefied to 50,000 reads per sample for downstream analysis.

### Statistical analysis

Alpha diversity indices of the endophytic fungi community, including richness index, Shannon-Wiener diversity index, Chao1 index, ACE index, and Simpson index, were calculated using the “vegan” package in R software (version 4.2.1). Principal coordinate analysis (PCoA) analyses based on Bray-Curtis distance were performed using the “vegan”, “microeco” and “ggplot2” packages in R software. Statistical differences among groups were assessed using one-way analysis of variance (ANOVA). When significant differences were detected, multiple comparisons were performed using Fisher’s Least Significant Difference (LSD) test. All analyses were conducted in R software using the aov function for ANOVA and the LSD.test function from the “agricolae” package. Co-occurrence patterns of endophytic fungi communities were constructed based on Spearman’s rank correlation coefficient. Co-occurrence events were identified as statistically robust correlations (|*r*|>0.6, *p* < 0.05), and the co-occurrence network was visualized in Gephi (version 0.10.1). Growth performance was evaluated using the following indices:

$$\:WGR\left(\%\right)=\frac{Final\:BW-Initial\:BW}{Initial\:BW\boldsymbol{}}*100$$

$$\:SGR\left(\%\right)=\frac{ln\left(Final\:BW\right)-ln\left(Initial\:BW\right)}{Days\boldsymbol{}}*100$$

$$\:BLGR\left(\%\right)=\frac{Final\:BL-Initial\:BL}{Initial\:BL\boldsymbol{}}*100$$

$$\:WGR\left(\%\right)=\frac{Final\:L-Initial\:L}{Initial\:L\boldsymbol{}}*100$$

where BW represents body weight (g), BL represents body length (cm), L refers to total length (cm), and Days is the duration of the feeding trial.

### Animal welfare statement

All experimental procedures involving animals were approved by the Ethical Committee for Experimental Animals of the Xizang Autonomous Region Academy of Agricultural and Animal Sciences, Institute of Aquatic Sciences (Approval No. 2023003). At the conclusion of the experiment, all fish were humanely euthanized in accordance with the AVMA Guidelines for the Euthanasia of Animals (2020) and the ARRIVE guidelines. Fish were first anesthetized in a buffered solution of tricaine methane sulfonate (MS-222; 150 mg/L) until loss of equilibrium and cessation of opercular movement were observed. After confirming deep anesthesia, euthanasia was completed using an overdose of MS-222 (300 mg/L) followed by pithing to ensure death. No fish regained consciousness during or after the procedure. All efforts were made to minimize animal suffering and stress throughout the study.

## Result

### Growth performance and hepatic antioxidant parameters of juvenile *Salmo trutta fario*

**Fig. 1:**
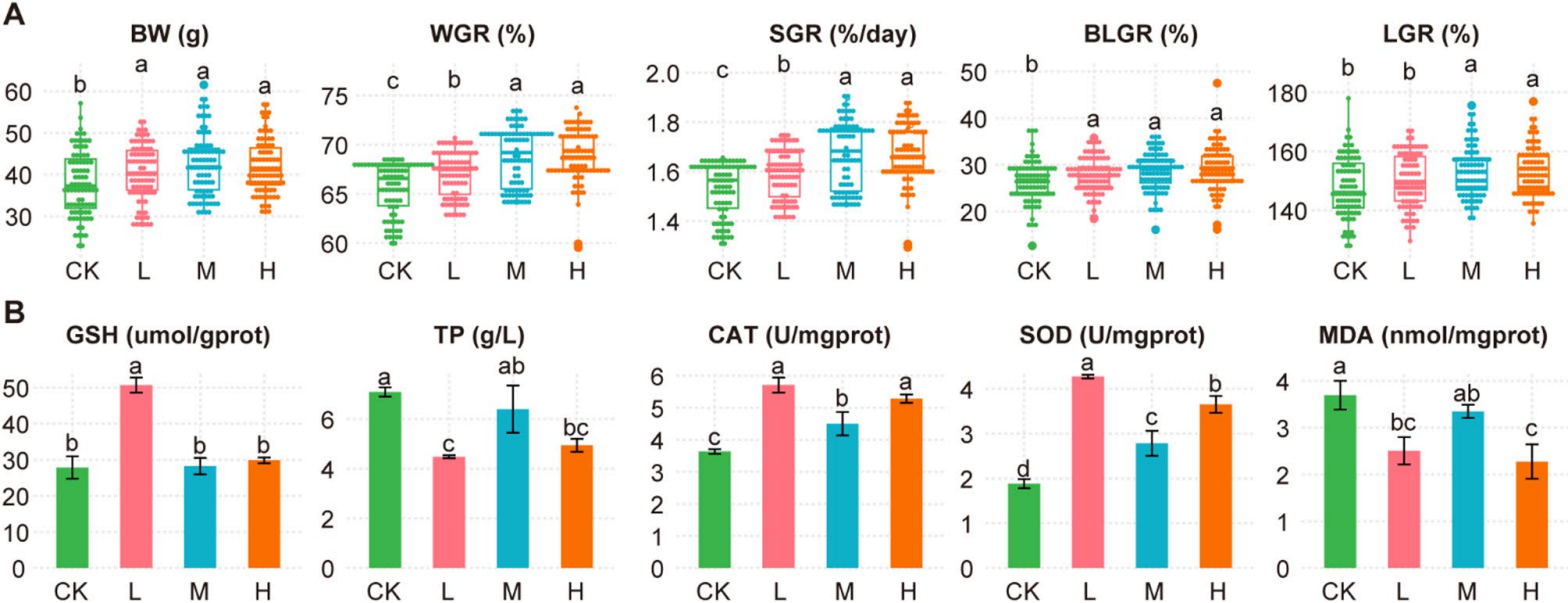
Growth performance and hepatic antioxidant parameters of Juvenile *Salmo trutta fario* under different probiotic treatments. (**A**) Growth performance indicators, including body weight (BW), weight gain rate (WGR), specific growth rate (SGR), body length growth rate (BLGR), and length gain rate (LGR), in four groups. (**B**) Hepatic antioxidant parameters including reduced glutathione (GSH), total protein (TP), catalase (CAT), superoxide dismutase (SOD), and malondialdehyde (MDA) in the four groups. Different letters above the bars indicate statistically significant differences between groups (*p* < 0.05).

Firstly, we analyzed the growth performance of Juvenile *Salmo trutta fario*. At the beginning of the experiment, there were no significant differences among the four groups in terms of body weight (BW), body length (BL), and standard length (L) (*p* > 0.05). After 70 days of feeding, the growth performance parameters are shown in Fig. 1A. For body weight (BW), the H group had the highest average value (41.85 g), while the CK group had the lowest (37.23 g). The BW values in the L, M, and H groups were significantly higher than that in the CK group (*p* < 0.05). The weight gain rate (WGR) was highest in the M group (69.20%) and lowest in the CK group (66.56%). WGR values in the L, M, and H groups were significantly higher than in the CK group. The specific growth rate (SGR) was also highest in the M group (1.68%/day) and lowest in the CK group (1.57%/day), with significant differences observed: the M group was significantly higher than the L group, and the L group was significantly higher than the CK group. The body length growth rate (BLGR) reached the highest value in the M group (29.08%) and the lowest in the CK group (26.77%), with all treatment groups (L, M, H) showing significantly higher BLGR than the CK group. The length growth rate (LGR) was highest in the M group (153.05%) and lowest in the CK group (146.06%), with both M and H groups being significantly higher than the CK and L groups. These results indicate that dietary supplementation with EM significantly improved the growth performance of Juvenile *Salmo trutta*, with the medium-dose group (M) showing the most pronounced effect. In addition, we measured the hepatic antioxidant parameters of Juvenile *Salmo trutta fario*, and the results are shown in Fig. 1B. GSH levels were significantly higher in the L group than in the other three groups. TP and MDA levels were significantly higher in the CK and M groups compared to the L and H groups. CAT activity was significantly higher in the L and H groups than in the M group, and the M group showed significantly higher CAT activity than the CK group. SOD activity showed significant differences among all four groups, following the order: L > H > M > CK. Notably, there were no significant differences in GSH, TP, and MDA between the M and CK groups, indicating that the hepatic antioxidant capacity of these two groups was relatively similar.

### Effects of dietary EM probiotics on hematological and serum biochemical parameters in juvenile *Salmo trutta fario*

**Fig. 2:**
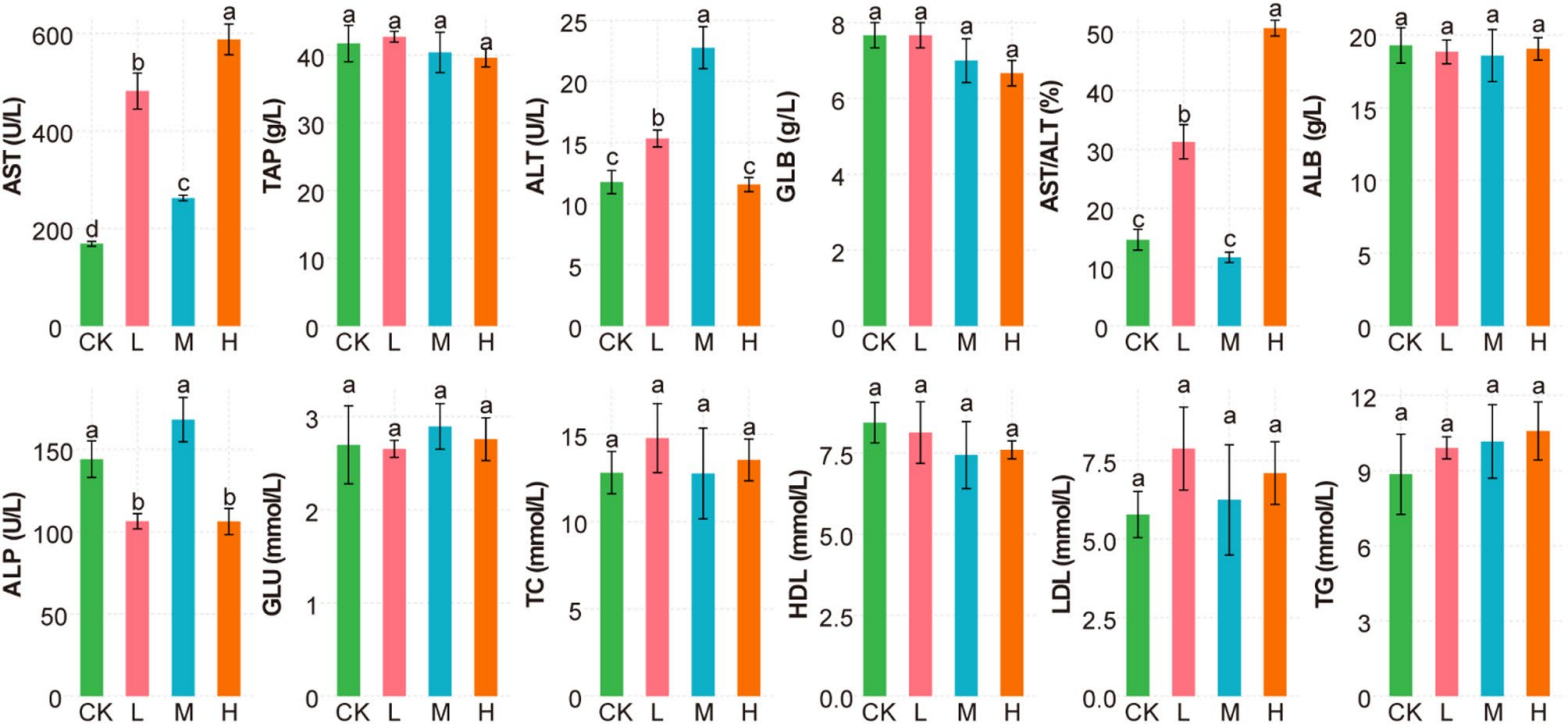
Effects of dietary EM probiotic supplementation on Serum Biochemical parameters in Juvenile *Salmo trutta fario.* Data are presented as means ± standard error (SE). Bars with different letters indicate significant differences among groups (*p* < 0.05, Tukey’s multiple comparison test). alanine aminotransferase (ALT), aspartate aminotransferase (AST), alkaline phosphatase (ALP), glucose (GLU), albumin (ALB), high-density lipoprotein cholesterol (HDL), low-density lipoprotein cholesterol (LDL), triglycerides (TG), and total cholesterol (TC). Data are presented as measured values (mean in parentheses). Data are presented as measured values (mean in parentheses).

To better understand the physiological status of Juvenile *Salmo trutta fario* we measured 12 Serum Biochemical parameters and conducted statistical comparisons among the four groups. As shown in Fig. 2, AST activity exhibited significant differences across four groups, with the highest value observed in group H (562.25), followed by groups L (454.64), M (263.18), and CK (166.86) in descending order. ALT activity was significantly higher in group M (22.39) compared to group L (15.26), and group L was significantly higher than both CK (11.90) and H (11.60) groups. The AST/ALT ratio was highest in group H (52.00), followed by L (32.00), CK (14.00), and M (12.00), showing statistically significant differences. ALP levels were significantly higher in groups CK (140.16) and M (155.91) compared to L (102.55) and H (106.93). Other hematological indices showed no significant differences among the groups.

**Fig. 3:**
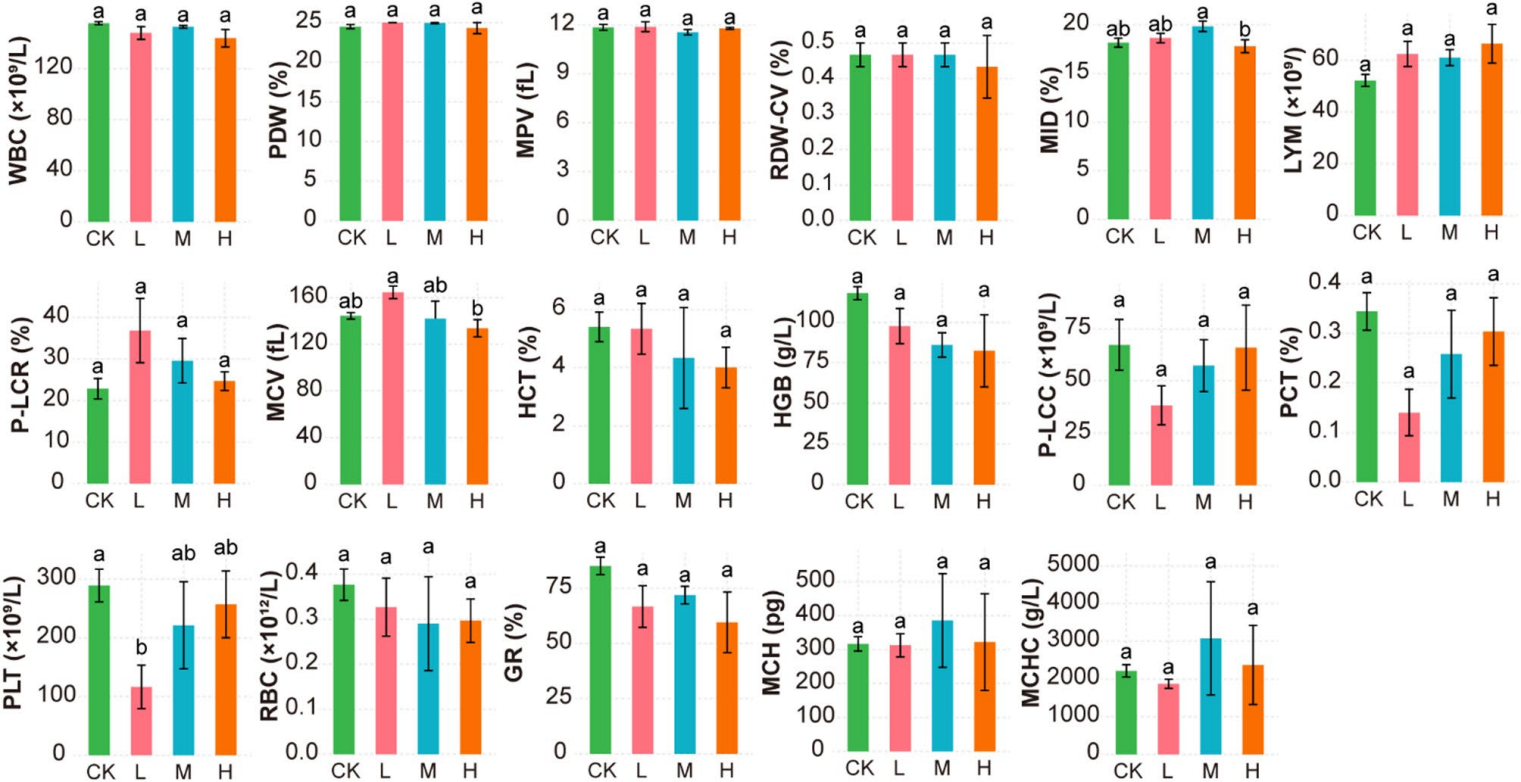
Effects of dietary probiotic supplementation on hematological parameters in Juvenile *Salmo trutta fario*. Values are expressed as mean ± standard error (SE). Different letters above bars indicate significant differences among groups (*p* < 0.05, Tukey’s test). white blood cell count (WBC), platelet distribution width (PDW), mean platelet volume (MPV), red cell distribution width-coefficient of variation (RDW-CV), Middle cell percentage(MID), Lymphocyte count (LYM), platelet large cell ratio (P-LCR), Mean Corpuscular Volume (MCV), hematocrit (HCT), hemoglobin (HGB), platelet large cell count (P-LCC), Plateletcrit (PCT), Platelet count (PLC), red blood cell count (RBC), granulocyte count (GR), mean corpuscular hemoglobin (MCH), mean corpuscular hemoglobin concentration (MCHC).

In addition, we analyzed hematological routine parameters, as shown in Fig. 3. MID were significantly higher in group M (13.30) than in group H (12.30), and MCV values were significantly higher in group L (162.20) than in group H (133.90). Platelet (PLT) counts were significantly higher in the CK (298.00) group compared to group L (115.00). No significant differences were observed in WBC, RBC, HGB, LYM, GR, P-LCR, P-LCC, HCT, MCHC, RDW-CV, MCH, MPV, PCT, and PDW across all groups (*p* > 0.05), suggesting that probiotic supplementation did not markedly alter overall leukocyte and erythrocyte profiles or platelet-related parameters.

### Diversity of intestinal microbiota communities under different levels of EM of juvenile *Salmo trutta fario*

**Fig. 4:**
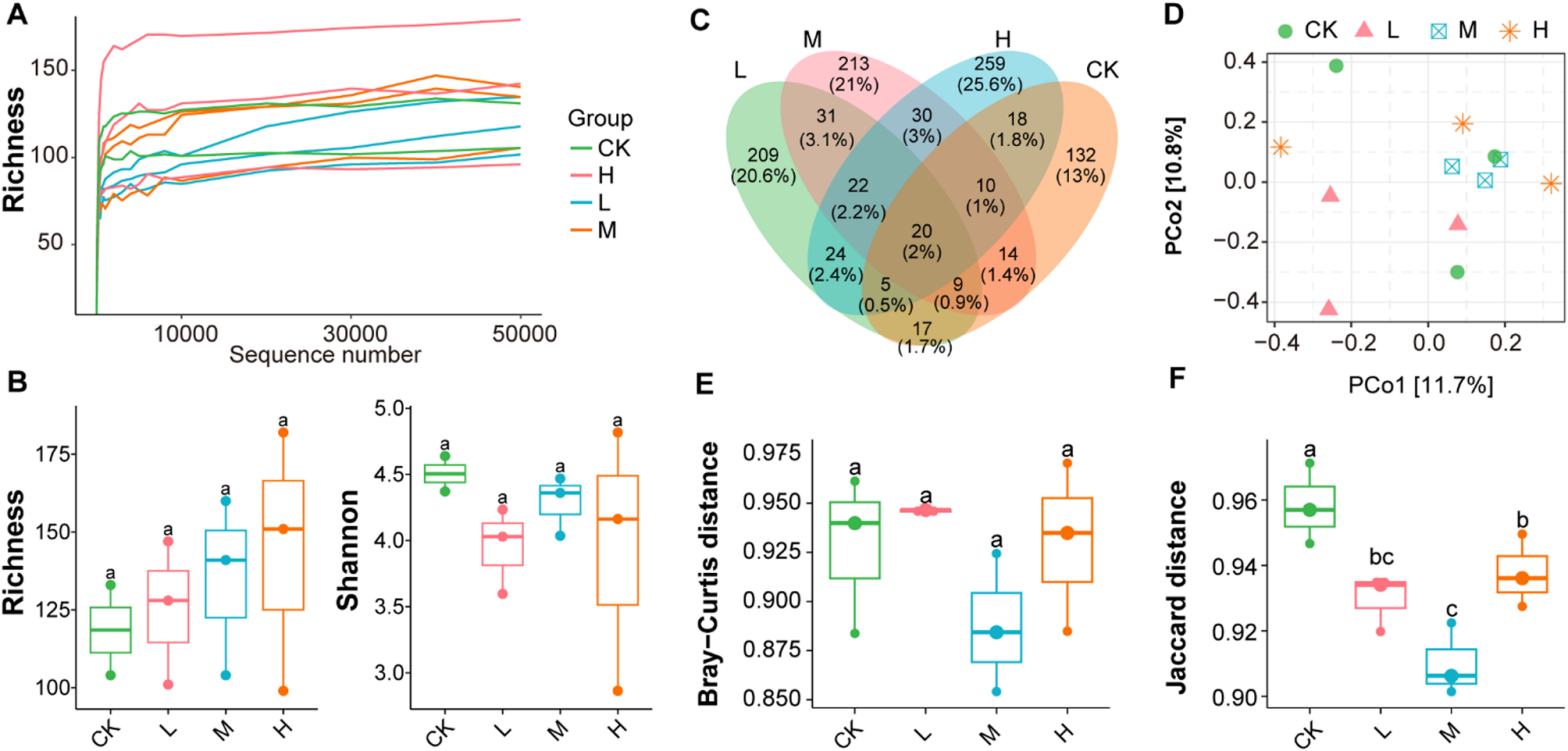
Diversity of the intestinal microbiota across the four groups. (**A**) Rarefaction curves of ASVs across all samples in each group. (**B**) Alpha diversity analysis based on richness and Shannon indices; differences among groups were not statistically significant. (**C**) Venn diagram showing shared and unique ASVs among the CK, L, M, and H groups. (**D**) Principal coordinate analysis (PCoA) based on Bray-Curtis distance illustrating beta diversity patterns among groups. (**E**) Boxplot of Bray-Curtis distances within each group. (**F**) Boxplot of Jaccard distances within each group. Statistical analysis was performed using one-way ANOVA followed by least significant difference (LSD) test for multiple comparisons. Different letters above the boxes indicate significant differences among groups at *p* < 0.05.

The diversity and richness of the intestinal microbiota were assessed across the three treatment groups and the control (CK) group. A total of 1739 amplicon sequence variants (ASVs) were detected. Specifically, 225 ASVs were identified in the CK group, 337 in the L group, 349 in the M group, and 388 in the H group. Rarefaction curves plateaued across all four groups, indicating sufficient sampling depth for the intestinal microbiota community (Fig. 4A). The sequencing effort was adequate to capture most of the microbial richness present in each group. The alpha diversity of the intestinal microbiota varied among groups based on different indices. For richness, values ranged from 104 to 133 (median: 118.5) in the CK group, 101 to 147 (median: 128) in the L group, 104 to 160 (median: 141) in the M group, and 99 to 182 (median: 151) in the H group. Overall, the median richness followed the trend H > M > L > CK; however, the differences were not statistically significant. For Shannon diversity, values ranged from 4.37 to 4.64 (median: 4.50) in the CK group, 3.60 to 4.23 (median: 4.03) in the L group, 4.04 to 4.47 (median: 4.36) in the M group, and 2.86 to 4.82 (median: 4.16) in the H group. The median Shannon index showed the trend M > CK > L > H, but again, the differences were not statistically significant (Fig. 4B). In addition, the Venn diagram was used to illustrate the shared and unique ASVs of the intestinal microbiota among the four groups (Fig. 4C). A total of 20 ASVs (2.0%) were shared across all groups. The CK group had 132 unique ASVs (13.0%), the L group had 209 (20.6%), the M group had 213 (21.0%), and the H group had 259 (25.6%). Furthermore, principal coordinate analysis (PCoA) was performed to evaluate beta diversity among the groups (Fig. 4D). The PCoA plot revealed that samples in the M group exhibited markedly lower beta diversity compared to the other groups. Consistently, both Bray-Curtis distance (Fig. 4E) and Jaccard distance (Fig. 4F) analyses showed that beta diversity in the M group was lower than in the other groups, with the Jaccard distance showing a statistically significant reduction. These results suggest that the intestinal microbiota composition in the M group was more stable, with less variation among samples.

### Composition of intestinal microbiota communities under different levels of EM of juvenile *Salmo trutta fario*

**Fig. 5:**
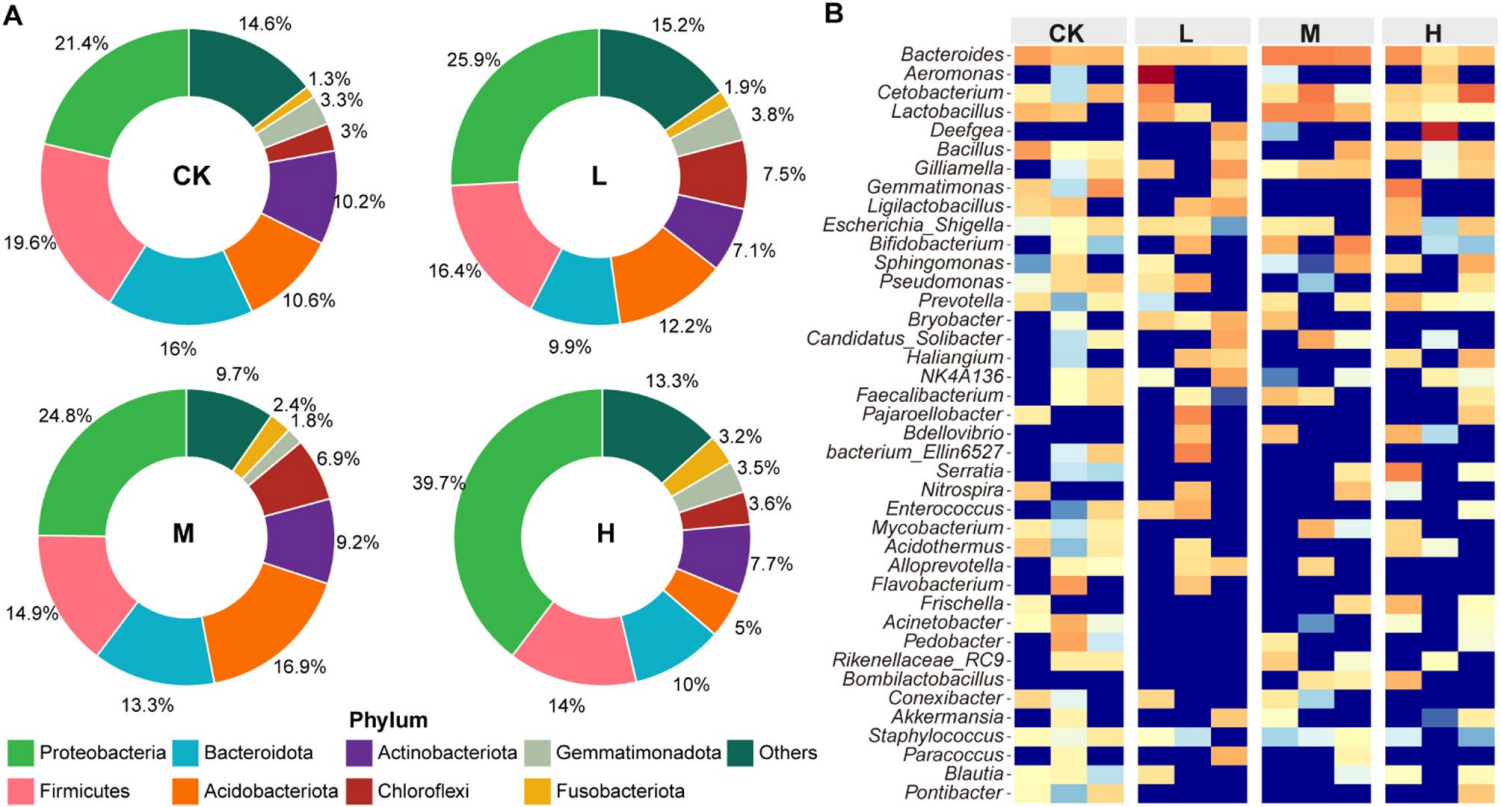
Composition of the intestinal microbiota across the four groups. (**A**) Relative abundance of dominant intestinal microbiota at the phylum level. (**B**) Relative abundance of dominant intestinal microbiota at the genus level.

In addition, we analyzed the dominant bacterial phyla of the intestinal microbiota in each group (Fig. 5A). In the CK group, the predominant phyla were Proteobacteria (21.4%), Firmicutes (19.6%), Bacteroidota (16.0%), Acidobacteriota (10.6%), Actinobacteriota (10.2%), unidentified (4.8%), Gemmatimonadota (3.3%), Chloroflexi (3.0%), Verrucomicrobiota (2.2%), and Myxococcota (1.6%). In the L group, the dominant phyla included Proteobacteria (25.9%), Firmicutes (16.4%), Acidobacteriota (12.2%), Bacteroidota (9.9%), Chloroflexi (7.5%), Actinobacteriota (7.1%), unidentified (4.3%), Gemmatimonadota (3.8%), Myxococcota (2.4%), and Verrucomicrobiota (2.3%). In the M group, the major phyla were Proteobacteria (24.8%), Acidobacteriota (16.9%), Firmicutes (14.9%), Bacteroidota (13.3%), Actinobacteriota (9.2%), Chloroflexi (6.9%), Verrucomicrobiota (2.5%), Fusobacteriota (2.4%), unidentified (2.3%), and Gemmatimonadota (1.8%). In the H group, the dominant phyla were Proteobacteria (39.7%), Firmicutes (14.0%), Bacteroidota (10.0%), Actinobacteriota (7.7%), Acidobacteriota (5.0%), Chloroflexi (3.6%), Myxococcota (3.6%), Gemmatimonadota (3.5%), and Fusobacteriota (3.2%).

At the genus level, the dominant genera of the intestinal microbiota varied across the four groups (Fig. 5B). In the CK group, the predominant genera included Bacteroides (2.5%), Gemmatimonas (1.7%), Bacillus (1.5%), Lactobacillus (1.3%), Acinetobacter (1.1%), Pseudomonas (1.1%), Flavobacterium (1.1%), Pedobacter (1.0%), and Cetobacterium (1.0%). In the L group, the dominant genera were Aeromonas (9.3%), Gilliamella (1.7%), Bryobacter (1.6%), Ligilactobacillus (1.6%), Bacteroides (1.5%), Pajaroellobacter (1.3%), Cetobacterium (1.3%), and Enterococcus (1.3%). In the M group, the major genera were Bacteroides (4.3%), Lactobacillus (3.5%), Bifidobacterium (2.1%), Cetobacterium (2.1%), Gilliamella (1.3%), Snodgrassella (1.1%), Candidatus Solibacter (1.1%), and Faecalibacterium (1.0%). In the H group, the dominant genera included Deefgea (5.3%), Cetobacterium (3.1%), Bacteroides (2.3%), Gemmatimonas (1.6%), Serratia (1.6%), Bacillus (1.3%), Escherichia-Shigella (1.3%), Sphingomonas (1.3%), and Haliangium (1.2%).

### Probiotics play important roles in the gut microbial co-occurrence network of *Salmo trutta fario*

**Fig. 6:**
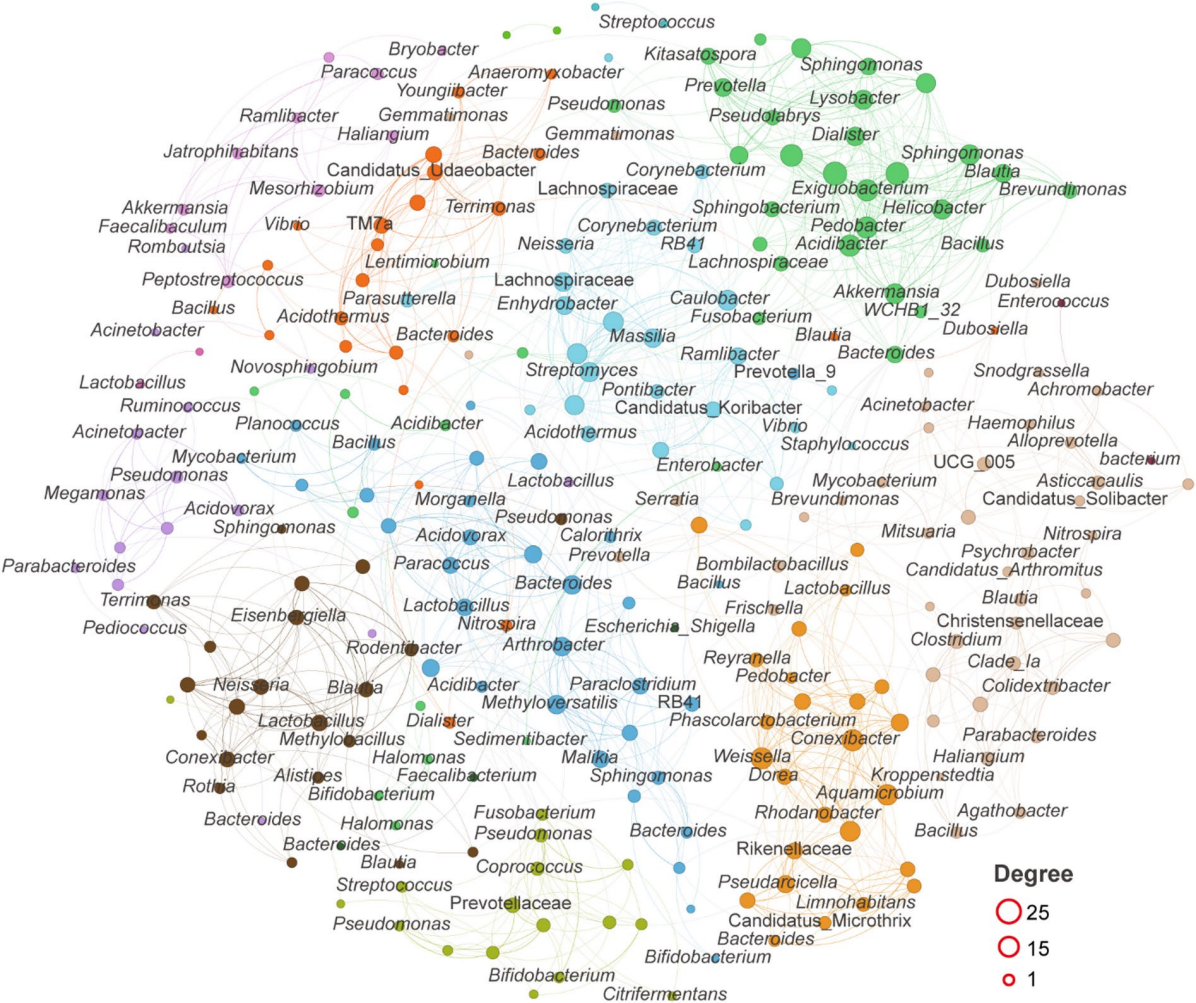
Co-occurrence network of intestinal microbial communities in Juvenile *Salmo trutta fario*. The network was constructed based on Spearman correlation coefficients (*r* > 0.6, *p* < 0.05) among bacterial ASVs. Each node represents ASV, and each edge indicates a significant correlation between ASVs. Different colors denote distinct network modules, and node size corresponds to the node degree.

To explore the potential interactions among intestinal microbial communities of *Salmo trutta fario*, a co-occurrence network was constructed, as shown in Fig. 6. The network comprised 267 nodes and 1188 edges, revealing complex interaction patterns among gut microbes. The modularity coefficient of the co-occurrence network was 0.83, which is greater than 0.4, indicating a clearly modular structure. In addition, we identified the top 20 genera with the highest degree values in the co-occurrence network, which may serve as potential keystone taxa. These genera include *Weissella*,* Conexibacter*,* Exiguobacterium*,* Pedobacter*,* Acidibacter*,* Aquamicrobium*,* Caulobacter*,* Akkermansia*,* Streptomyces*,* Lysobacter*,* Helicobacter*,* Methyloversatilis*,* Dialister*,* Arthrobacter*,* Bacteroides*, Lachnospiraceae_x, *Massilia*,* Blautia*, and *Enhydrobacter.* Among these genera, *Bacteroides*, *Weissella*, *Akkermansia*, *Blautia*, *Dialister*, *Exiguobacterium*, and *Massilia*, which are known or potential probiotic taxa, occupied keystone positions in the co-occurrence network. This suggests that these key genera may play important roles in regulating the intestinal microecology of Salmo trutta fario, contributing to the stability and functional diversity of the gut microbial ecosystem.

## Discussion

### Growth-promoting effects of probiotics on *Salmo trutta fario*

Probiotic supplementation in aquafeeds has been widely reported to promote fish growth and reduce susceptibility to disease^14,17,19,25^. Most previous studies have focused on single-strain probiotics^26,27^, whereas research involving compound or multi-strain probiotic mixtures remains relatively limited. Moreover, the dose-dependent effects of compound probiotics on growth performance, antioxidant capacity, and host–microbe interactions in salmonids have not been clearly defined. Our study contributes to this knowledge gap by systematically evaluating the physiological responses of juvenile *Salmo trutta fario* to different dietary levels of EM compound probiotics. In this study, we systematically evaluated the effects of different dietary levels of EM compound probiotics on the growth performance and serum biochemical parameters of juvenile *Salmo trutta fario*. Our findings confirmed that EM supplementation significantly enhanced growth performance, consistent with previous reports^24^. Among all treatments, the medium-dose group (M group, 0.5% EM) exhibited the most notable improvement, a pattern that aligns well with the results of Admasu et al.^28^, who also reported that moderate probiotic supplementation produced the most pronounced growth-enhancing effects. Specifically, the weight gain rate (WGR) in the M group reached 69.20%, which was 2.64% higher than the control, while the specific growth rate (SGR) increased to 1.68%/day, representing a 0.11% elevation compared to the control group. These results indicate that appropriate levels of EM supplementation can effectively promote growth and overall physiological development in juvenile *Salmo trutta fario*. We speculate that the growth-promoting effects of EM probiotics at optimal levels may be attributed to their ability to modulate the intestinal microbial environment and enhance nutrient absorption efficiency. Similar results have been reported by Serradell et al.^29^ who found that probiotic supplementation improves nutrient utilization and growth. Furthermore, Noveirian and Opiyo et al.^30,31^ reported that the addition of 0.3% Biogen probiotic in the diet significantly improved growth performance and feed utilization in juvenile carp, which is consistent with our findings. Therefore, this study provides further evidence supporting the growth-promoting potential of probiotics in aquaculture and underscores the importance of appropriate dosage levels to achieve optimal benefits^28^.

In addition to growth performance, we examined hepatic antioxidant capacity to better understand the physiological mechanisms underlying the beneficial effects of EM probiotics. The significant increases in GSH across all EM-treated groups suggest enhanced non-enzymatic antioxidant defenses, likely reflecting improved intracellular redox regulation. Previous studies have similarly reported that probiotics can elevate hepatic thiol levels and reduce oxidative injury^32,33^. The observed decreases in TP further imply reduced metabolic stress and improved liver homeostasis. The enzymatic antioxidant responses were also strongly modulated by EM supplementation. CAT and SOD activities were consistently elevated in EM-fed fish, indicating strengthened enzymatic scavenging of hydrogen peroxide and superoxide radicals, respectively. Comparable improvements in antioxidant enzyme activity have been reported in *Mugil capito*^33^, *Pangasianodon hypophthalmus*^32^, and *Micropterus salmoides*^34^, supporting the robustness of our findings. Meanwhile, the reduction in MDA levels suggests that EM probiotics effectively mitigated lipid peroxidation and oxidative membrane damage. Notably, MDA was not uniformly suppressed across all treatments, indicating that the antioxidant benefits may vary with dosage, and that excessively high probiotic inclusion may not confer additional protective effects^35^.

### Hematological and serum biochemical parameters in *Salmo trutta fario*

Hematological parameters in fish are valuable indicators for monitoring health status, as they are responsive to changes related to nutrition, water quality, disease, and therapeutic interventions^36,37^. Aspartate aminotransferase (AST) and alanine aminotransferase (ALT) are key biochemical indicators widely used to assess liver function and cellular damage in fish^38^. In the present study, serum biochemical and hematological indices were examined to clarify the physiological responses of *Salmo trutta* *fario* to different dietary levels of EM compound probiotics (Figs. 2 and 3). EM supplementation led to elevated AST activity across probiotic-treated groups, suggesting enhanced hepatic metabolic activity. This may be attributed to increased mitochondrial enzyme activity associated with amino acid metabolism, as previously reported in fish fed probiotic-enriched diets^39^. Interestingly, TP levels were significantly higher in the CK and M groups compared with the L and H groups. This pattern implies that moderate EM supplementation may support optimal plasma protein synthesis, whereas both insufficient and excessive probiotic doses might alter protein turnover efficiency or reduce stress-induced protein leakage. Such dose-dependent responses have also been observed in earlier studies, highlighting the importance of avoiding probiotic over-supplementation to prevent metabolic imbalance.

Hematological results further revealed that MID cell counts were significantly higher in the M group than in the H group, indicating that medium-dose EM probiotics may enhance the activity of specific innate immune cells involved in antigen processing and inflammatory regulation. This suggests a potential immunostimulatory effect at moderate supplementation levels, supporting a more robust immune response in juvenile *Salmo trutta*. Platelet numbers (PLT) were higher in the CK group than in the L group, yet platelet-associated parameters (P-LCR, PCT, PDW) showed no significant changes among treatments. These findings indicate that EM probiotics did not markedly influence blood coagulation function, hemostasis, or oxygen transport efficiency, which is consistent with the results reported by Ghori et al.^40^. Moreover, key leukocyte (WBC, LYM, GR) and erythrocyte (RBC, HGB, HCT) indices remained stable across all groups. The absence of significant alterations suggests that EM supplementation did not disrupt hematopoietic balance or systemic immune homeostasis, demonstrating its safety at the tested inclusion levels. Collectively, these results indicate that EM compound probiotics can modulate hepatic metabolism, antioxidant capacity, and innate immune activity to a certain extent, with the most beneficial effects observed in the medium-dose group. This aligns with reports that moderate probiotic doses provide improved physiological benefits compared with excessively low or high supplementation^41,42^.

### Effects of probiotics on gut microbial composition and diversity

The intestinal bacterial community is increasingly recognized as a key determinant of host metabolic status, immune regulation, and overall health, and probiotics can modulate these microbial structures and functions through competitive colonization, metabolite production, and niche modification^43,44^. In this study, we found that dietary supplementation with EM compound probiotics significantly altered the intestinal microbial composition of *Salmo trutta fario*, indicating that the host-microbe interface is highly responsive to probiotic dosage. Specifically, increasing levels of EM supplementation led to a rise in microbial richness (Fig. 4B). However, beta diversity decreased in the EM-treated groups, with the lowest beta diversity observed in the medium-dose group (Fig. 4E, F) suggesting that the intestinal microbial community of juvenile *Salmo trutta fario* was most stable under medium-dose EM supplementation. This pattern differs from the findings of Ghori et al.^40^, who reported that probiotic supplementation generally increases gut microbial alpha diversity. The abundance of core intestinal microbial phyla, including Proteobacteria, Firmicutes, Bacteroidota, and Actinobacteriota, was affected by different doses of EM supplementation (Fig. 5A). Firmicutes, often linked to lipid metabolism and promoted by lactic acid bacteria, showed a decreasing trend with higher EM doses, consistent with previous findings^20^. In contrast, Proteobacteria increased with EM dosage, indicating enhanced metabolic potential in the gut^45^. The Firmicutes/Bacteroidota (F/B) ratio, considered a marker of gut homeostasis and host growth^46^, was elevated in the M and H groups compared to the control, suggesting a possible link between EM supplementation and body weight gain. In addition, the co-occurrence network analysis of intestinal microbiota revealed that several probiotic genera, including *Weissella*, *Akkermansia*, *Blautia*, *Dialister*, *Exiguobacterium*, and Massilia, occupied keystone positions within the microbial interaction network. This suggests that these genera play central roles in maintaining intestinal microbial stability, and their increased centrality may partly explain the observed community compositional shifts under different EM supplementation levels^1,4^.

## Conclusion

This study systematically evaluated the effects of different dietary levels of EM compound probiotics on the growth, immune function, and intestinal health of juvenile *Salmo trutta fario*. The results demonstrated that EM probiotics significantly improved growth performance, with 0.5% supplementation demonstrating the most pronounced efficacy. In terms of hepatic antioxidant capacity, EM supplementation effectively enhanced non-enzymatic antioxidant defense, increased catalase (CAT) and superoxide dismutase (SOD) activities, reduced malondialdehyde (MDA) levels, and mitigated oxidative damage, thereby supporting liver health. Regarding hematological parameters, the medium-dose group exhibited significant improvements in immune regulation and protein metabolism. In terms of intestinal microbiota, EM probiotics altered the composition and diversity of the gut microbial community. The medium-dose group exhibited a more stable microbial structure. EM dosage influenced the abundance of key bacterial phyla such as *Firmicutes* and *Proteobacteria*, which are closely associated with host growth and immunity. Overall, dietary supplementation with 0.5% EM compound probiotics provided the most favorable effects on growth, antioxidant status, immune function, and intestinal microbial balance in juvenile *Salmo trutta*. These findings offer valuable insights for the development of efficient and healthy aquaculture strategies for salmonid species under high-altitude farming conditions.

## Data Availability

The raw sequence data reported in this paper have been deposited in the Genome Sequence Archive (Genomics, Proteomics & Bioinformatics 2021) in National Genomics Data Center (Nucleic Acids Res 2025), China National Center for Bioinformation / Beijing Institute of Genomics, Chinese Academy of Sciences (GSA: CRA028540) that are publicly accessible at https://ngdc.cncb.ac.cn/gsa/s/E5WJ30n3.
